# Recognition of Occluded Goods under Prior Inference Based on Generative Adversarial Network

**DOI:** 10.3390/s23063355

**Published:** 2023-03-22

**Authors:** Mingxuan Cao, Kai Xie, Feng Liu, Bohao Li, Chang Wen, Jianbiao He, Wei Zhang

**Affiliations:** 1 School of Electronic and Information, Yangtze University, Jingzhou 434023, China; 2 National Electrical and Electronic Experimental Teaching Demonstration Center, Yangtze University, Jingzhou 434023, China; 3 Western Research Institute, Yangtze University, Karamay 834000, China; 4 School of Computer Science, Yangtze University, Jingzhou 434023, China; 5 School of Computer Science and Engineering, Central South University, Changsha 410083, China

**Keywords:** intelligent retail, recognition of occluded goods, Generative Adversarial Network (GAN), semantic inference, feature expansion, vMF distribution

## Abstract

Aiming at the recognition of intelligent retail dynamic visual container goods, two problems that lead to low recognition accuracy must be addressed; one is the lack of goods features caused by the occlusion of the hand, and the other is the high similarity of goods. Therefore, this study proposes an approach for occluding goods recognition based on a generative adversarial network combined with prior inference to address the two abovementioned problems. With DarkNet53 as the backbone network, semantic segmentation is used to locate the occluded part in the feature extraction network, and simultaneously, the YOLOX decoupling head is used to obtain the detection frame. Subsequently, a generative adversarial network under prior inference is used to restore and expand the features of the occluded parts, and a multi-scale spatial attention and effective channel attention weighted attention mechanism module is proposed to select fine-grained features of goods. Finally, a metric learning method based on von Mises–Fisher distribution is proposed to increase the class spacing of features to achieve the effect of feature distinction, whilst the distinguished features are utilized to recognize goods at a fine-grained level. The experimental data used in this study were all obtained from the self-made smart retail container dataset, which contains a total of 12 types of goods used for recognition and includes four couples of similar goods. Experimental results reveal that the peak signal-to-noise ratio and structural similarity under improved prior inference are 0.7743 and 0.0183 higher than those of the other models, respectively. Compared with other optimal models, mAP improves the recognition accuracy by 1.2% and the recognition accuracy by 2.82%. This study solves two problems: one is the occlusion caused by hands, and the other is the high similarity of goods, thus meeting the requirements of commodity recognition accuracy in the field of intelligent retail and exhibiting good application prospects.

## 1. Introduction

With the expansion of the internet in recent years, purchasing and consumption have become more convenient. For example, vending machines may be employed in a variety of complicated applications owing to their low labor costs, compact footprint, low running expenses, and high income per unit. The rapid development of consumer poverty alleviation vending machines is a concrete practice for consolidating and expanding the achievements of poverty alleviation and rural revitalization; additionally, it is consistent with the new tendency of “non-touch commerce” in the face of the epidemic [1,2,3]. Traditional vending cabinets are based primarily on the radio frequency identification (RFID) [4] and static object detection [5] technologies; however, they tend to have significant manufacturing and maintenance expenses. Dynamic and smart vision-based cabinets, as opposed to traditional cabinets, integrate computer vision and cloud computing to complete the purchasing process of customers, with precise recognition of commodities being one of its most significant and key functions. The entire purchasing process is recorded by a camera to recognize the classes of commodities in the users’ hands [6]; however, because of the varying degrees of occlusion brought by the actions of manual picking up of commodities, the ability of convolutional neural network to extract features for commodities is limited, which results in inaccurate recognition results.

Two main issues need to be resolved to recognize commodities with occlusion caused by hand and occasion when the features of goods are highly similar: 1. the lack of commodity features owing to occlusion caused by human hands, thus necessitating restoration and expansion; 2. the low recognition accuracy due to the high degree of similarity of some products, which dictates the need to distinguish between similar features.

To address these issues, this study proposes an algorithm for goods recognition under occlusion based on prior inference and spherical clustering. First, generative adversarial network (GAN) is combined with semantic inference whilst appropriate noise priors are matched with pre-trained generators and noise predictors. Next, a Hausdorff distance-based contour structure loss function is used during the training process to render the process of feature expansion more effective and accurate. Then, an attention mechanism is utilized to select the most discriminative fine-grained features for the expanded features. Subsequently, the von Mises–Fisher (vMF) distribution [7] is utilized to map the expanded and selected fine-grained features onto a unit hypersphere for clustering to increase the spacing of features. During the process, an angle loss is designed to improve the effect of clustering, and finally, the softmax function is utilized to obtain the ultimate recognition results.

## 2. Related Work

### 2.1. Conventional Recognizing Method

Several algorithms have been used to recognize smart retail commodities. The YOLO series [8] and SSD [9] are typical examples of single-stage algorithms that combine detection with classification tasks into a single stage. Faster-region-based convolutional neural network (Faster-RCNN) [10] and cascade R-CNN [11] are examples of multi-stage algorithms that divide feature extraction and regression tasks into two stages. Recognition of objects and faces under occlusion has advanced rapidly in recent years, thus yielding novel ideas and approaches for recognizing occluded commodities [12,13,14], which are broadly classified into two classes, one of which is adding weight to the unoccluded part and another is restoring the occluded part. Kortylewski et al. [15] proposed a compositional convolutional neural network (CNN) model to recognize products based on unoccluded parts. Wang et al. [16] proposed an object shape feature extraction approach called slope difference distribution (SDD), which extracts features of shape as a sparse representation and utilizes the detected SDD features of all shape models and the minimum distance between SDD features for object recognition. Ma et al. [17] proposed a robust face recognition approach based on a sparse network with limited probability, built a sparse image network with limited probability, and acquired the overall training images from a global perspective for recognition. Heo et al. [18] proposed an occlusion-aware spatial attention transformer (OSAT) architecture based on a visual transformer (ViT), CutMix strengthening, and occlusion mask predictor (OMP) to solve the occlusion problem. Xu et al. [19] proposed a double-active-layer-based CNN to recognize facial expressions with high accuracy by learning robust and discriminative features from data. However, because of the occlusion caused by the hand and the great resemblance of the commodities, the accuracy of goods recognition using conventional methods is low.

### 2.2. Recognizing Based on Feature Expansion Method

Although the network topologies and approaches described above can successfully improve the recognition of items and faces under occlusion, the recognition accuracy remains low because of the lack of goods features. A GAN [20] is an adversarial game-based neural network that can be utilized to generate missing features of commodities. Arjovsky et al. [21] proposed Wasserstein GAN (WGAN), which addresses the difficulties in gradient disappearance and collapse during the training of GAN networks. Gulrajani et al. [22] proposed the WGAN-gradient penalty (WGAN-GP) to boost the convergence speed of networks. Liao et al. [23,24] proposed a semantic guidance and evaluation network (SGE-Net) to iteratively update the structural prior and restore images in an interactive framework of semantic extraction and image restoration; additionally, they designed a semantic wise attention propagation (SWAP) module to restore the integral details of texture. Li et al. [25] proposed a recursive feature reasoning (RFR) network to restore the largely missing textures of images. However, feature expansion and restoration can not address the problem of high similarity of goods.

### 2.3. Recognizing Based on Fine-Grained Method

Feature restoration and expansion are excellent solutions to the problem of the small number of product features; however, their similarity to other commodities is a crucial challenge for the recognition of comparable commodities. Geng et al. [26] proposed an approach for the recognition of fine-grained commodities based on feature matching and one-time deep learning to handle the issue of high feature similarity. Lee et al. [27] proposed a network that combines a linear model with a deep learning model, considering both discrete features and the content of continuous images. Rao et al. [28] proposed a counterfactual attention learning approach based on causal inference to concentrate on fine-grained features through counterfactual intervention. Wang et al. [29] proposed an improved fine-grained classification approach based on self-attention destruction and constructive learning (SADCL). Liu et al. [30] proposed a scale-consistent attention part network (SCAPNet) for fine-grained image recognition. Although the fine-grained recognition accuracy for unoccluded goods is high, it cannot achieve its best efficiency for the recognition of goods under occlusion.

## 3. Methods

**Figure 1:**
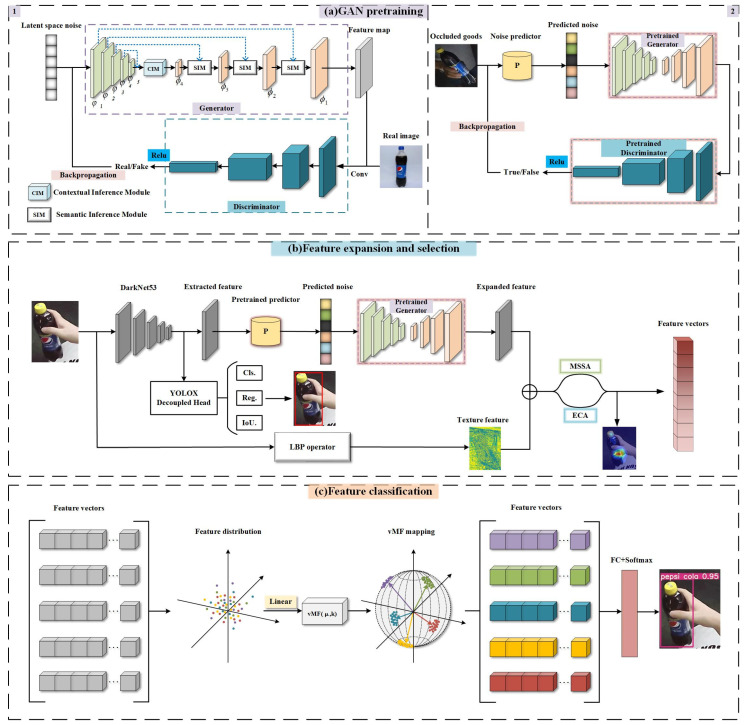
Algorithm flow of the proposed architecture for goods recognition. The numbers 1 and 2 in the **upper left** and **upper right** corners represent the first and second parts of GAN pretraining.

Figure 1 shows the flowchart of the overall algorithm in this study. The algorithm includes three elements, namely GAN pretraining, feature expansion and selection of commodities, and features classification of commodities. (1) The GAN model and parameters are trained with the addition of a semantic inference module and noise prior to increase the accuracy and efficiency of the generated features. (2) The pre-trained GAN is utilized for the restoration and expansion of features of the occluded parts and then combines them with LBP features [31]. Simultaneously, the attention mechanism is utilized to select features with a greater degree of discrimination after expansion. (3) Metric learning based on vMF distribution is utilized to distinguish and classify among selected features.

### 3.1. GAN Pretraining

Regarding the similarity of object recognition, every time a new object is under recognition when the network is trained, the recognition efficiency of the processing is low. To increase the accuracy and efficiency of the entire recognition process, all the images of commodities in the library are sent to the GAN for pre-training, during which each input image is matched with an appropriate latent space noise distribution [32], which in turn is utilized in the actual recognition process of commodities under occlusion. The predicted noise prior is used for the following process of feature restoration and expansion to guarantee the accuracy of the generated features.

#### 3.1.1. Generator Pretraining

**Figure 2:**
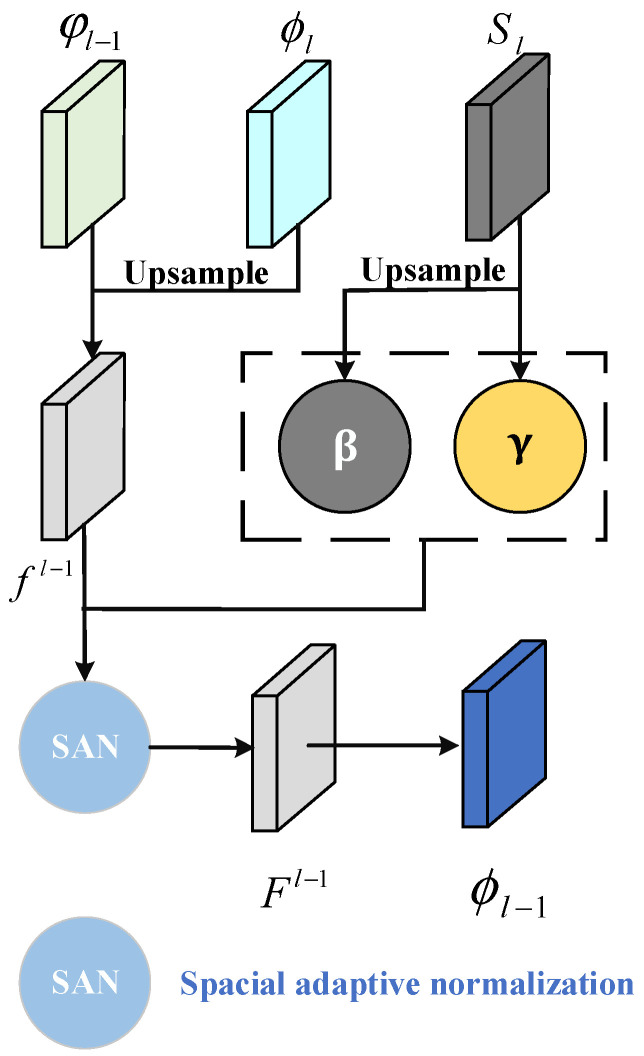
Semantic Inference Module. The current encoding feature $\phi_{l}$ and decoding features $\varphi_{l-1}$ are sent to SIM to be fused with a skipping connection.

In the network training process, the generator input is set as random latent spatial noise [33] and a commodity feature map is generated during the overall process. After completing the iterative training, the generator parameters are saved and await feature expansion of the commodity in the next process. The generator is a neural network with an encoder–decoder architecture, where the encoder is associated with five dense blocks in sequence, whereas the decoder is associated with four dense blocks and three semantic inference modules (SIM) connected crosswise in sequence. The encoder and the decoder are linked using a context inference module (CIM). Each dense block of the encoder comprises three layers of batch normalization, an activating function layer (LeakyReLU), and convolution layer (Conv2D), wherein the corresponding size of the convolution kernel in the convolution layer is 3 × 3. Padding is not utilized in the convolution layer, and the alpha value of LeakyReLU is 0.01. Each dense block is followed by a downsample layer made up of a 1 × 1 convolution layer and a 2 × 2 pooling layer to halve the size of the feature map. The encoded contextual features are sent to the CIM for the inference of contextual features, after which the encoded feature map is sent for decoding. The structure of the four dense blocks in the decoder is identical to those of the encoder. Each dense block is followed by an oversampling layer composed of a 1 × 1 convolution layer and transposed convolution layer, which doubles the size of the feature map. The SIM process is illustrated in Figure 2.

The current encoding feature $\phi_{l}$ and decoding features $\varphi_{l-1}$ are simultaneously sent to SIM wherein they are first fused with a skipping connection, as follows:

(1) $$f^{l-1}=\sum_{i=1}^{C}\left(\varphi_{l-1}+\phi_{l}⊗K_{i}^{T}\right)⊗K_{i},$$

where $K_{i}$ and $K_{i}^{T}$ are the convolution kernels on the i-th channel and its transpose, respectively; and ⊗ represents convolution, l ∈ 1,2,3,4.

Next, semantic segmentation is implemented on the image to acquire a semantic segmentation map $S_{l}$. This allows for the implement spatial adaptive normalization on its parameters to acquire the updated inferred features:

(2) $$F^{l-1}=\gamma ⊙\frac{f^{l-1}-\max\left(f^{l-1}\right)}{\max\left(f^{l-1}\right)-\min\left(f^{l-1}\right)}+\beta ,$$

where γ and β are parameters in the semantic segmentation map $S_{l}$, with γ representing the normalized scaling coefficient and β is the term of bias; and ⊙ representing the corresponding multiplication of matrix elements. 

**Figure 3:**
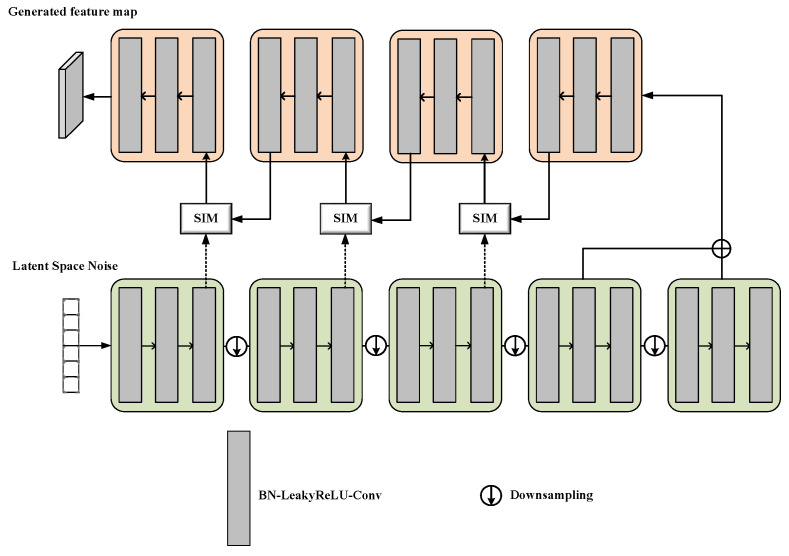
Structure of generator.

The generator and discriminator constantly learn from each other, and the generator parameters are fixed when reaching the Nash equilibrium. The network structure of the generator is illustrated in Figure 3.

#### 3.1.2. Discriminator Pretraining

The discriminator constantly learns from the generator to increase the recognition accuracy of the sample. Herein, the CNN was utilized in the discriminator training process, with the real and feature maps generated by the generator as the inputs, to determine whether the generated feature map was suitable for a real distribution. Additionally, dimensionality reduction was accomplished using a convolutional layer with a step size of 2, convolutional kernel size of 3 × 3, activation function of LeakyReLU, dropout operation to avoid overfitting, parameter *p* = 0.2, and output layer activated by the Tanh activation function. The fully linked layer outputted the final discriminant result.

#### 3.1.3. Noise Prior Pretraining

In this study, the precision of the feature expansion was improved by training the predictor to match the precise prior noise during the feature-generating process. First, the images of the occluded commodities were input, and the predictor was utilized to match the appropriate noise prior to guiding the generator for the generation of correct features. These are highly similar to the known features of commodities and thus ensure the accuracy of the generated features. The parameters were fixed and utilized for the expansion of commodity features after numerous rounds of iterative training.

#### 3.1.4. Loss Function

Through an adversarial game involving generator *G* and discriminator *D*, the GAN optimizes the value function *V*(*G*,*D*) as follows:

(3) $$\underset{G}{\min}\underset{D}{\max}V\left(D,G\right)=E_{x∼p_{r}\left(x\right)}\left[\log D\left(x\right)\right]+E_{z∼p_{g}\left(z\right)}\left[\log\left(1-D\left(G\left(z\right)\right)\right)\right],$$

where *z* is the random noise input in the latent space; *G(z)* denotes the generated feature matrix; *D*(*x*) denotes the probability that the discriminator’s judgment of the generated features of the generator is true; $p_{r}\left(x\right)$ denotes the distribution of the real features; and $p_{g}\left(z\right)$ denotes the distribution of the generated features.

The JS divergence between $p_{r}\left(x\right)$ and $p_{g}\left(z\right)$ was optimized to maximize the training effect of *G* and *D* in the game between the generator and discriminator to reach $p_{r}\left(x\right)$ = $p_{g}\left(z\right)$. However, the gradient vanishing phenomenon often occurs during the training process of the GAN, thereby resulting in the divergence of JS being a constant and producing a gradient vanishing issue. The similarity index of the two distributions is calculated using the Wasserstein distance combined with the Sobolev constraint [34], which is more versatile compared with the Lipschitz constraint.

The Wasserstein distance of the Sobolev constraint is

(4) $$\underset{G}{\min}\underset{D}{\max}L_{s}\left(D,G\right)=E_{x∼p_{r}}D\left(x\right)-E_{z∼p_{g}}D\left(G\left(z\right)\right).$$

The constraint condition is

(5) $$E_{x∼f^{x_{i},x_{j}}}\|\nabla_{x}D{\left(x\right)\|}^{2}-1\leq 0,x_{i}∼p_{r},x_{j}∼p_{g},$$

where $f^{x_{i},x_{j}}\left(x\right)$ is the probability density function of random variable x between $x_{i}$ and $x_{j}$. Assuming that t is a random variable obeying a uniform distribution between [0, 1], the linear interpolation of the real sample $x_{i}$ and generated sample $x_{j}$ is

(6) $$x=tx_{i}+\left(1-t\right)x_{j}.$$

Then, $f^{x_{i},x_{j}}\left(x\right)$ can be represented as

(7) $$f^{x_{i},x_{j}}\left(x\right)=\{\begin{array}{l} \int_{x_{i}}^{x_{j}}\frac{1}{\sqrt{2\pi }\sigma }e^{-\frac{{\left(x-\mu \right)}^{2}}{2\sigma^{2}}}dx\\ 0,\;\mathrm{otherwise}\; \end{array},x=tx_{i}+\left(1-t\right)x_{j},$$

where *x* is the linear interpolation between the real sample $x_{i}$ and the generated sample $x_{j}$; and μ and σ (σ > 0) are the mean and standard deviation of the Gaussian distribution, respectively.

For *D*(*x*) to satisfy the Sobolev constraint, the gradient penalty *GP* was used, which is defined as

(8) $$GP=E_{x∼p_{x}}\left[{\left(\|\nabla_{x}D{\left(x\right)\|}_{2}-1\right)}^{2}\right].$$

When discriminating the contours of generated features and real features, a contour structure loss function called Hausdorff Distance loss was designed using the Hausdorff Distance model, which is more sensitive to the contour structure. The pixel points on the shallow contour feature map of the network were viewed as a set of k pixel points, where the contour of the feature map extracted from the real image was $C_{r}=\left\{A_{1},A_{2},\ldots ,A_{k}\right\}$, and the goods’ contour of the generated feature map was $C_{g}=\left\{B_{1},B_{2},\ldots ,B_{k}\right\}$. The directional Hausdorff Distance of the contours of the goods in the two images can be expressed as

(9) $$h\left(C_{r},C_{g}\right)=\underset{A_{k}\in C_{r}}{\max}\underset{B_{k}\in C_{g}}{\min}\|A_{k}-B_{k}\|,$$

(10) $$\begin{array}{c} h\left(C_{g},C_{r}\right)=\underset{B_{k}\in C_{g}}{\max}\underset{A_{k}\in C_{r}}{\min}\|B_{k}-A_{k}\| \end{array}.$$

The coordinates of the pixel points in the real and generated feature maps correspond to each other.

Thus, the Hausdorff Distance of the coordinates is

(11) $$H\left(C_{r},C_{g}\right)=max\left\{h\left(C_{g},C_{g}\right),h\left(C_{g},C_{r}\right)\right\}.$$

Therefore, the contour structure loss function $L_{Hausdorff\;distance}$ is

(12) $$L_{Hausdorff-distance}=H\left(C_{r},C_{g}\right).$$

Consequently, the loss function of the discriminator in this study is

(13) $$\underset{D}{\min}L_{s}\left(G,D\right)=E_{x\sim p_{r}}\left[D\left(x\right)\right]-E_{x\sim p_{g}}\left[D\left(x\right)\right],\;\;\;\;\;\;\;\;\;\;\;\;\;\;+\lambda E_{x\sim f^{x_{i},x_{j}}}[\left(\|\nabla_{x}D\left(x\right){\|}_{2}-1{)}^{2}\right]+\alpha H\left(C_{r},C_{g}\right),$$

where $P_{g}$ is the distribution of the generated feature map; $P_{r}$ is the distribution of the real feature map; x represents the linear interpolation between the real sample $x_{i}$ and the generated sample $x_{j}$; and λ is the weight coefficient, which is updated during the iteration process and taken as λ = 8, α = 4.

To guarantee the consistency between the generated and real features during the training process of the generator, the generator was trained iteratively using the combined loss of adversarial loss $L_{1}$ and gradient loss $L_{2}$.

If only the contour structure loss function is available in the discriminator, the generator can guarantee only the generation of accurate coarse contours but not clear texture features. Therefore, an adversarial loss function was added, and its expression is

(14) $$L_{1}=\underset{D}{\max}\left[\log D\left(\left(x_{r}\right)\right)+\log(1-D\left(x_{g}\right)\right)],$$

where $x_{r}$ denotes the real feature distribution; and $x_{g}$ denotes the generated feature distribution.

The $L_{1}$ loss function ensures that the generated texture features are accurate; however, in the back-propagation process of the generated features, ensuring the feature coherence of the generated feature maps is challenging. Therefore, this study introduced the gradient loss function $L_{2}$ to ensure coherence between the generated features and the real features, as follows:

(15) $$L_{2}=\underset{F_{r}\cup F_{g}}{\iint }\left|\left|\nabla F_{g}\left(x,y\right)\right|\right|dxdy,$$

where $F_{r}$ denotes the real feature map; $F_{g}$ denotes the generated feature map; and $\left|\left|\nabla F_{g}\left(x,y\right)\right|\right|$ denotes the pixel gradient modulus of the point (*x*, *y*) in the generated feature map.

Thus, the loss function of the generator is as follows:

(16) $$L_{g}=\beta L_{1}+\gamma L_{2},$$

where β and γ are weight coefficients.

The overall objective function is as follows:

(17) $$\underset{G}{\min}\underset{D}{\max}L\left(G,D\right)=E_{x\sim p_{r}}\left[D\left(x\right)\right]-E_{x\sim p_{g}}\left[D\left(x\right)\right],\;+\lambda E_{x\sim f^{x_{i},x_{j}}}[\left(\|\nabla_{x}D\left(x\right){\|}_{2}-1{)}^{2}\right],\;+\alpha \max\{h\left(C_{r},C_{g}\right),h\left(C_{g},C_{r}\right)\},\;+\beta \underset{D}{\max}\left[\log D\left(\left(x_{r}\right)\right)+\log(1-D\left(x_{g}\right)\right)],\;+\gamma \underset{F_{r}\cup F_{g}}{\iint }\left\|\nabla F_{g}\left(x,y\right)\right\|dxdy,$$

where λ, α, β, and γ are the weight coefficients used to guarantee that the loss functions are balanced.

### 3.2. Expansion and Selection of Features

When recognizing similar occluded commodities, the number of features of the commodities typically determines the recognition accuracy. When a human hand picks up a product and creates different degrees of occlusion, the effective features available for recognition are reduced, and the commodity’s effective features need to be restored and expanded to increase the accuracy of commodity recognition. This study utilized a pre-trained GAN, semantic inference module, and predictor P to expand the number of commodity features.

#### 3.2.1. Three-Channel Feature Extraction

When a commodity is occluded manually, using only a single gray channel to extract the features results in poor commodity recognition accuracy. Consequently, in this study, the recognition accuracy was improved by increasing the number of effective features of the commodity by extracting the features of goods using red–green–blue (RGB) three-channel feature extractor.

First, the OpenCV library was used to intercept the frame of the product image from a video stream photographed; subsequently, DarkNet53 was utilized as the backbone network to extract and concatenate the RGB three-channel feature map. That is,

(18) $$F^{*}=\left[\sum_{i=1}^{c}F^{i}\right]⊗K,$$

where $F^{*}$ is the feature map after feature fusion; *c* = 3 is the number of channels; $F^{i}$ is the feature map on the *i*-th channel; and *K* is a 1 × 1 convolution kernel.

**Figure 4:**
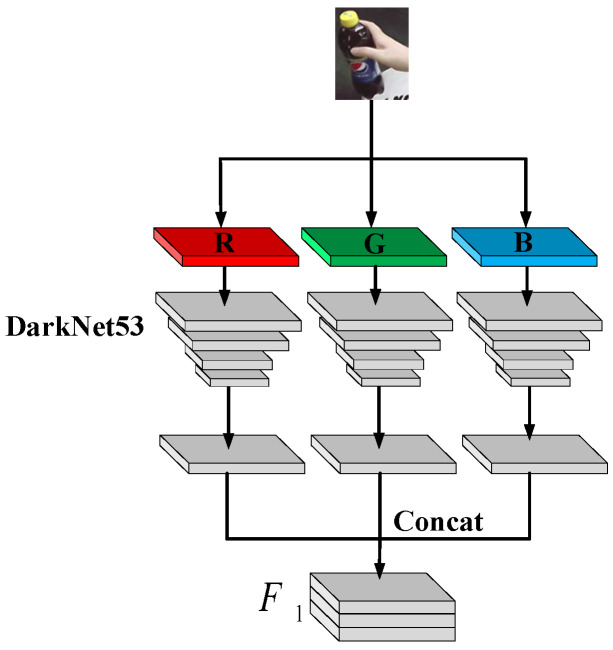
Feature extraction of RGB three-channel.

The flow of feature extraction of RGB three-channel is shown in Figure 4.

#### 3.2.2. Feature Restoration and Expansion

Herein, targeting the situation where the goods have few valid features owing to being occluded by hands, a combination of generative adversarial networks and semantic inference was used to extract relevant detailed features such as contours and textures.

First, the LBP operator was used to extract the texture features of the image of a good as it excels at describing the local texture features of the goods. Second, the previously extracted image features were sent to a pre-trained predictor (P) that predicts the latent spatial noise prior to adjusting its feature distribution. Finally, feature-matched noise was utilized to guide the expansion of features using a pre-trained generator.

#### 3.2.3. Features Selection

Given that a large variety and number of features are provided, which is not beneficial for recognizing similar goods, herein, a self-designed lightweight attention mechanism, multi-scale spatial attention (MSSA), combined with effective channel attention (ECA), which focuses on the most distinctive features of the feature map and selects them in a short period, was used.

In MSSA, a 1 × 1, 2 × 2, and 3 × 3 convolution kernels were used to extract the multi-scale features to gain a feature pyramid, and the discrimination scores were calculated. The higher the discrimination score, the greater the distinction between features. The calculation process is as follows:

(19) $$\mathrm{score}=W_{2}\cdot \mathrm{SiLU}\left(W_{1}G\left(x\right)\right),$$

where *G* denotes global average pooling; $W_{1}$ and $W_{2}$ are different full connection layers; and the SiLU activation function was used to enhance the ability of non-linear activation.

Thus, the attention feature vectors were obtained through the discrimination score pyramid under spatial pyramid pooling (SPPF).

**Figure 5:**
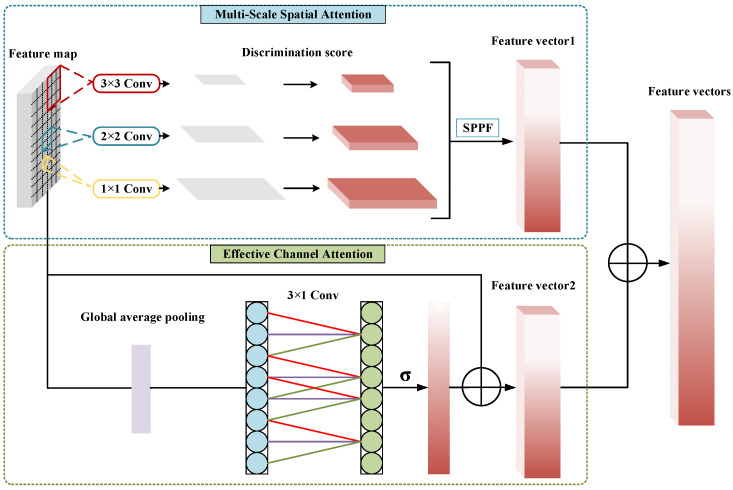
Flow of features selection.

In ECA, first, pooled feature maps were acquired utilizing global average pooling; subsequently, a 3 × 1 fast convolution was utilized and the attention feature map was acquired using a sigmoid activation function. Next, the expanded features were fused with the attentional feature map, and the feature vectors under MSSA and ECA were weightedly fused to acquire the final selected features of goods. A flowchart of the feature selection is presented in Figure 5.

### 3.3. Features Distinction

The high similarity among features in the process of similar goods recognition under occlusion is the most significant problem that must be resolved. Herein, first, the similarities were classified at a finer scale to increase the spacing of the selected fine-grained features, and then the most identifiable features were selected to further improve the recognition accuracy. Further, metric learning based on the vMF distribution was used and a continuous probability distribution that is a Gaussian distribution on a sphere which is a distribution function for modeling high-dimensional spaces was used to increase the spacing of features. The accuracy and effectiveness of the classification can be improved by mapping the feature vectors to the unit hypersphere, treating them as feature points on the sphere, and then clustering them. This increases the separation among various classes of features, which provides each class with more distinct classification boundaries.

#### 3.3.1. Feature Mapping and Clustering

Herein, the feature vectors were mapped onto the hypersphere using vMF distribution. The feature vector *x* must conform to the vMF distribution on the sphere if it conforms to the Gaussian distribution and the probability density function, which can be expressed as

(20) $$f\left(x|\mu ,k\right)=c_{p}\left(k\right)e^{k\mu^{T}x},$$

where μ,k are the mean vector and aggregation parameters, both of which parameterize the vMF distribution, respectively; and $c_{p}\left(k\right)$ is the normalization constant, denoted as

(21) $$c_{p}\left(k\right)=\frac{k^{p/2-1}}{{\left(2\pi \right)}^{p/2}I_{p/2-1}\left(k\right)},$$

where p is the number of variables and $I_{p/2-1}\left(k\right)$ denotes the P/2−1 first- class modified Bessel function.

The vMF clustering centers were determined by maximizing the vMF likelihood of the training feature vectors. The vMF likelihood is defined as

(22) $$p(f_{j}|\lambda )=\frac{e^{\sigma \mu^{T}f_{i}}}{Z\left(\sigma \right)},\left|\left|f_{j}\right|\right|=1,\left|\left|\mu \right|\right|=1,$$

where μ is the mean value of the vMF distribution; σ is the standard deviation; λ={μ,σ}; Z(σ) is the normalization constant; $f_{j}$ is the feature vector; i is the number of cluster centers; and $f_{i}$ is the cluster centroid feature vector.

Note that the loss function must be minimized to maximize the vMF probability; moreover, the feature vector $f_{j}$ is assumed to be assigned to the vMF clustering center $\mu_{i}$ during training. In the clustering process, first, a random point was chosen from the hyperspherical feature points as the centroid of the first class of features; subsequently, the corresponding loss function was set to classify the nearest k points into one class. Here, k is the aggregation parameter, which represents the number of feature points clustered close to the centroid. Next, the second centroid point was determined, and so on, until the spherical feature points were clustered into n classes to complete the overall clustering process.

**Figure 6:**
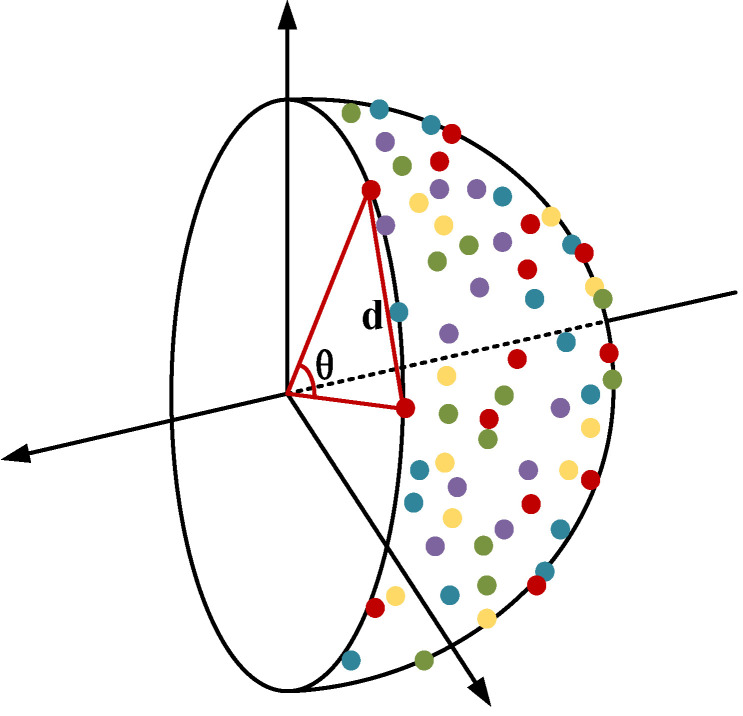
Cross-section of features on unit hypersphere. Different colored points represent feature vectors of different classes.

Given that the spacing between the features is in the form of arcs as they are situated on a sphere and move around the sphere as they cluster, their distances cannot be calculated using only the Euclidean distance. Therefore, the distances between the feature vectors and center vectors were quantified according to the angle θ between them, and an angle-based loss function was constructed to constrain the entire clustering process. Figure 6 shows a cross-section of the unit hypersphere feature.

The following is the process of derivation. According to the cosine theorem,

(23) $$\cos\theta =\frac{\sum_{i}^{N}\left(x_{i}{}^{2}+y_{i}{}^{2}\right)-d^{2}}{2\sqrt{\sum_{i}^{N}x_{i}{}^{2}}\cdot \sqrt{\sum_{i}^{N}y_{i}{}^{2}}},$$

where $\left(x_{1},x_{2},\ldots ,x_{N}\right),\left(y_{1},\ldots ,y_{N}\right)$ denotes two random feature vectors with N dimensions on the hypersphere; and d denotes the Euclidean distance between them.

Given that the features lie in the unit hypersphere, the radius is 1, which yields

(24) $$\theta =\arccos\frac{2-d^{2}}{2}.$$

The angle-based loss function $L_{\theta }$ can be defined as

(25) $$L_{\theta }=\arccos\frac{2-\sum_{n=1}^{N}{\left(x_{i\_n}-x_{j\_n}\right)}^{2}}{2},$$

where $\left(x_{i\_1},x_{i\_2},\ldots ,x_{i\_N}\right)$ are the coordinates of the *i*-th cluster center on the unit hypersphere of dimension N, and the size of N depends on the dimension of the feature vectors; and $\left(x_{j\_1},x_{j\_2},\ldots ,x_{j\_N}\right)$ are the coordinates of the jth feature vector on the unit hypersphere of dimension N. Minimizing $L_{\theta }$ enables better clustering of the k points closest to the clustering center.

In the process of finding the (*i* + 1)-th centroid, a probability formula was used, defined as follows:

(26) $$p=\frac{d{\left(f_{i},f_{j}\right)}^{2}}{\sum_{f_{j}\in F}d{\left(f_{i},f_{j}\right)}^{2}},$$

where $f_{i}$ is the *i*-th cluster center feature point on the hypersphere; and $f_{j}$ is the *j*th feature point on the hypersphere.

Under the constraint of the loss function, feature points are constantly clustered to form n classes of features on the hypersphere, with each class consisting of k feature points.

#### 3.3.2. Feature Classification

The softmax loss function is normally used for the classification of features. After inputting the final feature vectors of the fully connected layer, the result is normalized, the input features are converted into the form of probability, and the one with the highest probability score is the final result of classification. Unlike the general softmax loss function, this study used spherical softmax loss which can better describe the distribution of feature points on the sphere and classify them.

This loss function can be defined as

(27) $$L_{s}=-\frac{1}{N}\sum_{i=1}^{N}\log\frac{e^{s\cos\theta_{yi}}}{\sum_{j=1}^{C}e^{s\cos\theta_{j}}},$$

where s is the scale factor and s = 5; *N* is the number of feature points; C is the number of categories; $\theta_{yi}$ is the angle between the weight matrix of the sample labels and the feature vector $x_{i}$; and $\theta_{j}$ is the angle between the weight matrix of the jth class of sample labels and the feature vector $x_{j}$.

For the final classification, the fine-grained characteristics that were clustered and mapped onto the hypersphere were fed into the softmax function, and the results of goods detection under occlusion were output.

## 4. Experimental Results and Analysis

To evaluate the performance of the proposed algorithm in feature restoration and product recognition accuracy, an ablation experiment and a comparative experiment are set up, and the performance of the algorithm is evaluated according to the experimental data.

### 4.1. Dataset

**Figure 7:**
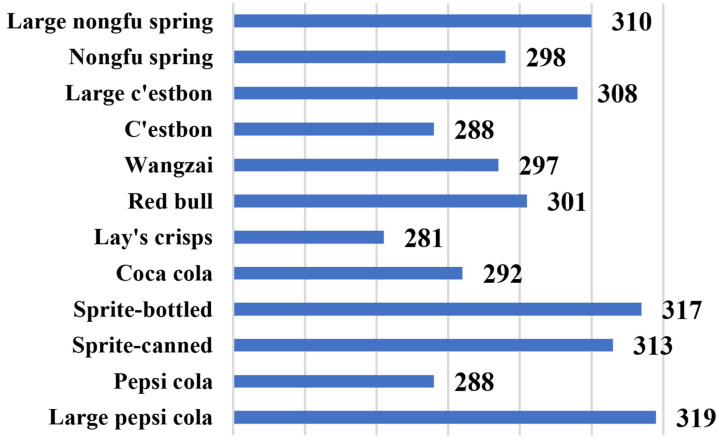
The number of each kind of good in our dataset.

**Figure 8:**
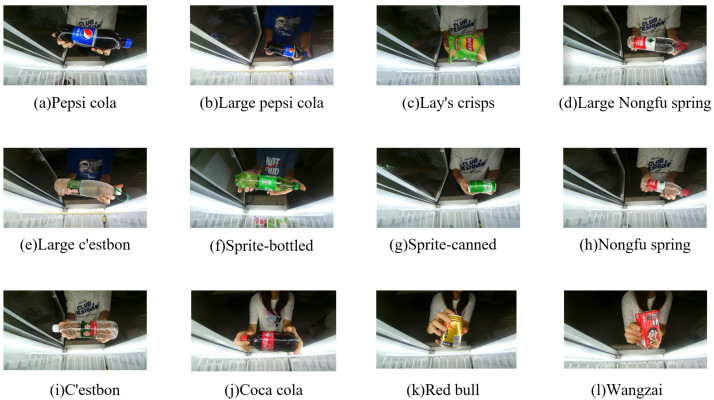
Some of the pictures in our dataset. Below these images are the names of these products.

Owing to the complex situation of smart retail containers in practical applications, at present, only commodities appear in the open-source dataset, with no character interaction or alternative complex environments; therefore, the recognition accuracy of the algorithm in the actual application process cannot be guaranteed. To ensure that commercial standards can be reached in the actual operation process, the experimental data used in this study were all obtained from the self-made smart retail container dataset, which contains a total of 12 types of goods, including canned, bagged, and bottled goods. By filming the entire process from opening the door to picking up the product and closing the door, the video frame of the product picture from each angle was captured for training. Among them, the lighting conditions were normal indoor lighting and the background was dark owing to the light strip. Figure 7 shows the number of images for each product in the dataset. Figure 8 shows some of the pictures in our dataset.

### 4.2. Experiment Platform

**Figure 9:**
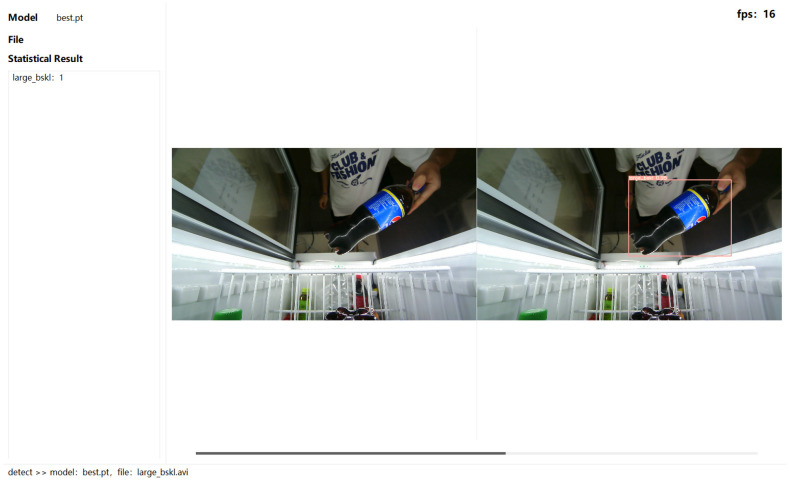
The UI interface of proposed method.

The experimental platform included an operating system Windows 10, graphics card NVIDIA GeForce RTX3060, processor AMD Ryzen 7 5800H with Radeon Graphics. The Pytorch1.11 deep neural network framework and Python3.7 were used to build the network models. Figure 9 shows the UI Interface of proposed method.

### 4.3. Feature Restoration and Expansion Result

Note that owing to the occlusion caused by the hand when purchasing goods, some of the features of goods are missing, thus causing a decrease in recognition accuracy. Therefore, feature restoration and expansion were utilized to increase recognition accuracy. The following experiments were conducted to evaluate the effects of feature restoration and expansion. Further, a self-made dataset was utilized for feature restoration and expansion, in which a mask was used to simulate the occlusion caused by the hand.

#### 4.3.1. Ablation Experiment

To justify the effectiveness of the Hausdorff Distance applied in the loss function of the discriminator in the proposed feature restoration and expansion algorithm in this study, a video frame during the user picking up the goods was shot and the ablation experiment was performed with the loss function in WGAN and WGAN-GP, which are the peak signal-to-noise ratio (PSNR) and structural similarity index measure (SSIM). After several debugging and optimization cycles, the parameters in the code were set with 300 epochs, a batch size of 32, Adam optimizer, learning rates of the generator and discriminator as 0.0001 and 0.0004, respectively, and the hyperparameters $\beta_{1}$ and $\beta_{2}$ as 0.5 and 0.9, respectively. The results of the experiments are listed in Table 1.

#### 4.3.2. Comparison Experiment

**Figure 10:**
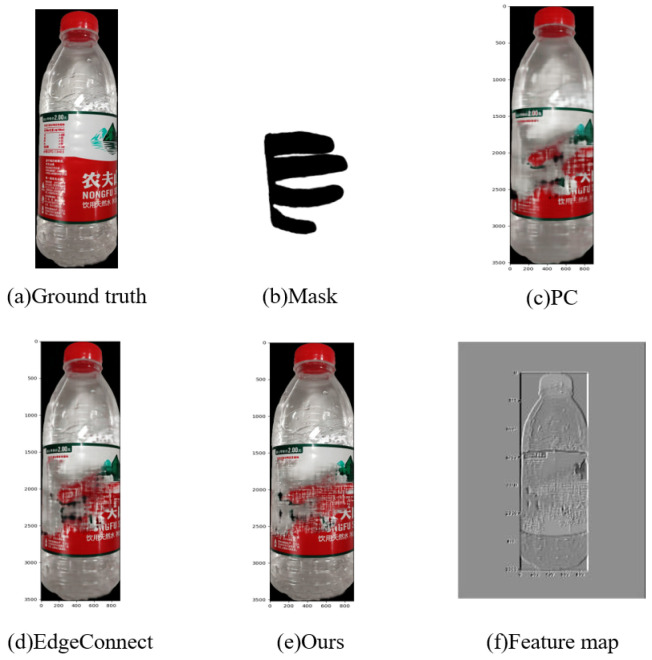
Comparison experiment of feature restoration and expansion.

To evaluate the capability of the Semantic inference module applied in generative adversarial network for feature restoration and expansion, the commonly utilized image inpainting algorithms partial convolution (PC) [35] and edge connect [36] were compared with the proposed method. Convolution visual evaluation was used to evaluate the effect of feature restoration and expansion, with experimental results indicating that the proposed method is superior to the abovementioned methods. The experimental results are shown in Figure 10.

### 4.4. Recognition Results of Occluded Goods

#### 4.4.1. Ablation Experiment

To evaluate the effectiveness of prior GAN and spherical clustering in the occluded goods recognition algorithm in this study, a neural network ablation experiment based on DarkNet53 was performed, and its capability was evaluated using the F1 score and mean average precision (mAP).

(28) $$F1-\mathrm{Score}=2\times \frac{\mathrm{precision}\times \mathrm{recall}}{\mathrm{precision}+\mathrm{recall}},$$

(29) $$\mathrm{mAP}=\frac{\sum_{n=1}^{N}\mathrm{AveP}\left(n\right)}{N},$$

where N denotes the number of queries; and AveP(n) denotes the average precision.

(30) $$\mathrm{precision}=\frac{\mathrm{TP}}{\mathrm{TP}+\mathrm{FP}},$$

(31) $$\mathrm{recall}=\frac{\mathrm{TP}}{\mathrm{TP}+\mathrm{FN}},$$

where TP denotes the positive samples predicted to be positive by the model; FN denotes the positive samples predicted to be negative by the model; and FP denotes the negative samples predicted to be positive by the model.

After debugging and optimization, the parameters in the code were set as 100 epochs, batch size 8, Adam optimizer, a learning rate of 0.0001, and the hyperparameters $\beta_{1}$, $\beta_{2}$ were both equal to 0.999. The following experiments are based on the parameters used to train the neural networks and the experimental results are as Table 2. Although both our F1 score and mAP are superior to other combinations, the optimal parameters remain to be explored.

**Figure 11:**
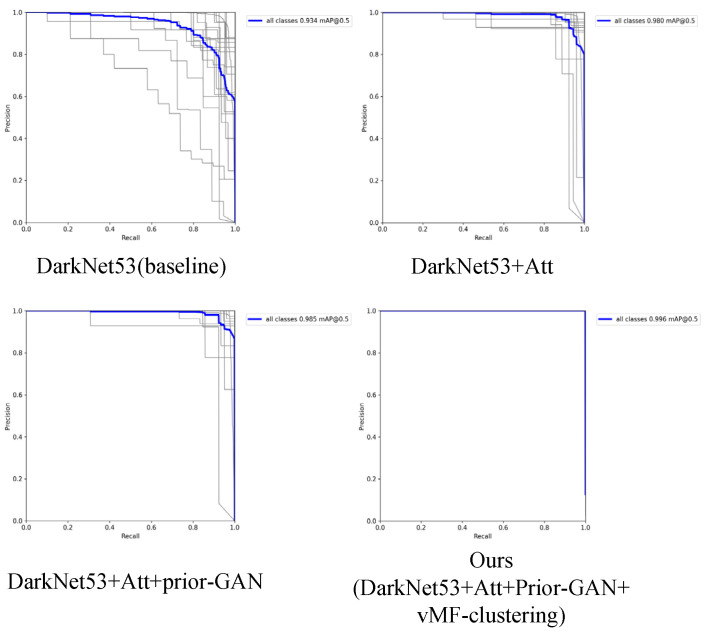
Ablation experiment of P–R curve under different combinations of networks.

To evaluate the recognition performance of various neural network combinations on the dataset, the P–R curve, denoting the precision–recall curve, was utilized. In theory, the larger the area enclosed by the P–R curve and the horizontal and vertical axes, the stronger the capability of the algorithm. Results demonstrate that the P–R curve of the proposed method is superior to the following network combinations. The experimental results are shown in Figure 11.

#### 4.4.2. Comparison Experiment

**Figure 12:**
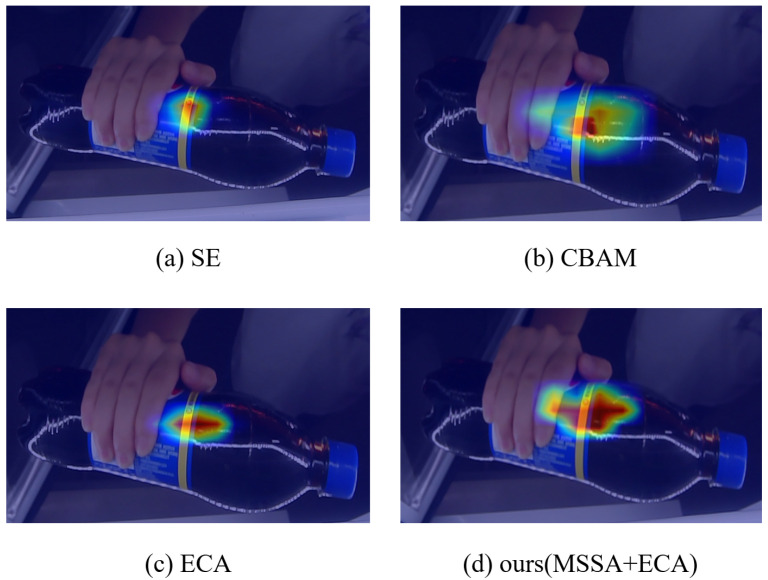
Heatmaps of various attention mechanism algorithms.

To evaluate the effectiveness of feature selection under the proposed attention mechanism, that is, ECA + MSSA, a comparison experiment based on squeeze excitation (SE) [37], convolutional block attention module (CBAM) [38], and ECA [39] was conducted to compare the attention heatmap with the proposed method. The experimental results are shown in Figure 12, with the proposed method achieving the best performance.

To justify the improvement of proposed attention mechanism on increasing the recognition accuracy, the effects of the proposed attention mechanism were compared with other attention mechanisms through recognition accuracy; that is, the actual videos of the purchasing process were recognized and the recognition results of each frame of the videos were averaged such that the class corresponding to the highest confidence was the ultimate recognition result of this purchasing process. Recognition accuracy denotes the number of correctly recognized videos divided by the total number of videos. The recognition accuracy in the following experimental results was based on the standard. The experimental results, including the F1 score, mAP, and recognition accuracy, are listed in Table 3.

**Figure 13:**
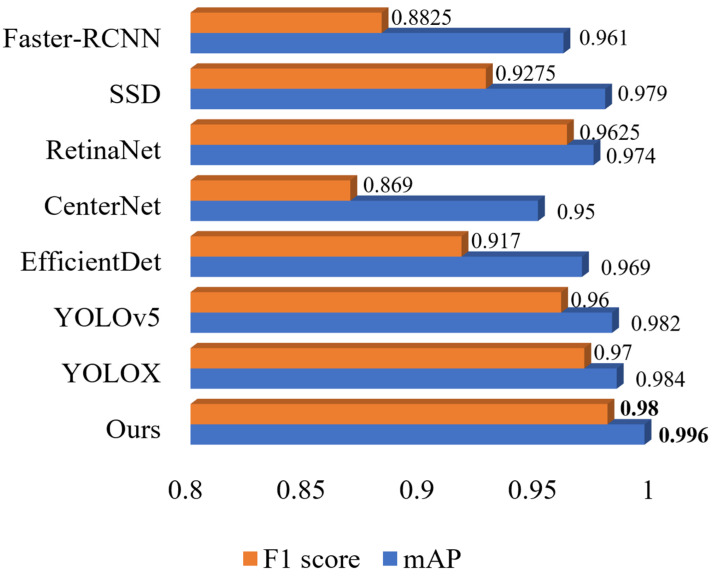
Comparison experiment of training parameters on various algorithms. Note: Bold is the best result.

To evaluate the proposed algorithm for occluded goods recognition, several mainstream object detection algorithms were compared with the proposed algorithm, and the F1 score and mAP of each algorithm during the training process were recorded for comparison. The experimental results are shown in Figure 13.

**Figure 14:**
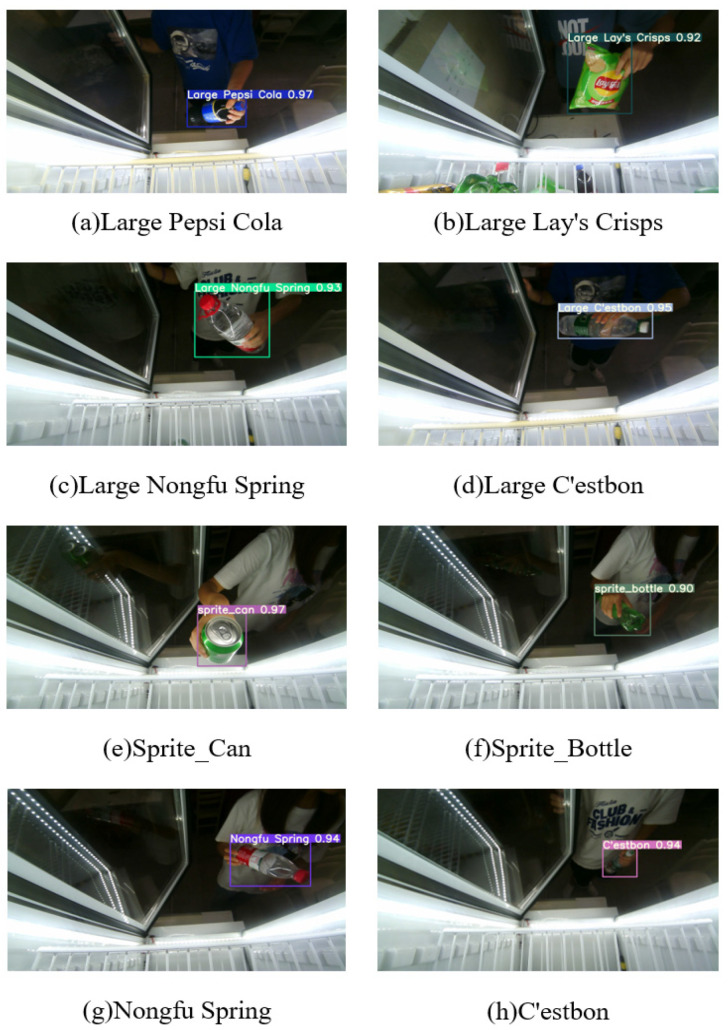
Diagrams of recognition results on various scenarios and persons.

During the process of good recognition, a detection frame was drawn on the detected goods, and on this detection frame, the class and corresponding confidence were marked. A schematic is shown in Figure 14, which shows different scenarios and the purchasing process based on different persons.

To evaluate the generalization capability of various object recognition algorithms, the smart retail goods were divided into three shapes and the videos of each shape of goods were recognized in the actual purchasing process. The training effect of each algorithm reached an optimal state on the same dataset. The recognition accuracy results are presented in Table 4. The approach in this paper achieved good results, but there is little progress in the recognition accuracy of canned goods, which needs improvement.

To reflect the improvement of the proposed algorithm on the recognition accuracy of occluded similar goods, a comparison experiment comprising four groups of similar goods on various algorithms was designed. The recognition accuracy of each good was recorded, as summarized in Table 5. For similar goods recognition, Table 5 shows that the proposed approach performs well in most cases, but still has shortcomings in a small number of cases such as in recognizing “Sprite_Bottled” and “Little Nongfu Spring”, which needs improvement.

## 5. Conclusions

This study proposed an algorithm for the recognition of occluded goods under prior inference and spherical clustering, which resolves not only the problem of few features of goods caused by hand occlusion but also the occasion when the features of goods are highly similar.

1. To solve the problem of the small number of good features due to occlusion, the generator and predictor of the network were pretrained, and noise was used prior to guide the generation of features. By jumping and connecting context features for semantic inference, these features could be restored and expanded. The contour structure loss function based on the Hausdorff Distance designed in this study was used in the process of feature restoration and expansion to ensure the generation of accurate contour features.
2. To resolve the situation in which the product features are highly similar, MSSA+ECA was used to select the features in a fine-grained manner and the most discriminative features were screened out. The spherical model under the vMF distribution was used to map the feature vectors to the unit hypersphere and cluster it, thereby increasing the distance between features. In the spherical feature clustering process, the angle loss function designed in this study was used to effectively improve the clustering effect.

The experimental results in this study achieved good subjective and objective evaluation results, thus proving the feasibility and effectiveness of the proposed method. However, the proposed algorithm must be optimized and improved. Given that feature restoration and clustering require a certain amount of time and the consumption scenarios are complex, the real-time performance and generalization ability of the algorithm need to be improved in future research. Additionally, the algorithm process can be further explored to improve real-time and generalization capabilities to deal with more complex situations. In the future, the algorithm can be applied to the unmanned smart vending method of goods in shopping malls, supermarkets, and other places to provide a reliable and good recognition method for the smart retail field.

## Figures and Tables

**Table 1:** Ablation experiment of loss function.

Loss Function	PSNR	SSIM
$Loss_{GAN}+loss_{Wasserstein}$	19.0742	0.7367
$Loss_{GAN}+loss_{Wasserstein-GP}$	19.2039	0.7481
Ours ^1^	**19.9782**	**0.7664**

^1^ Ours ($loss_{GAN}+loss_{Wasserstein-GP}+loss_{Hausdorff}$). Note: Bold is the best result.

**Table 2:** Parameters of ablation experiment of neural network.

Backbone	F1 Score	mAP
DarkNet53 (baseline)	0.94	0.934
DarkNet53 + Attention	0.95	0.980
DarkNet53 + Pr-GAN + Attention	0.95	0.985
Ours	**0.98**	**0.996**

Note: Bold is the best result.

**Table 3:** Parameters of comparison experiment of attention mechanism.

Network Combinations	F1 Score	mAP	Accuracy
DarkNet53 (baseline)	0.94	0.934	0.894
DarkNet53 + SE	0.95	0.980	0.907
DarkNet53 + CBAM	0.96	0.983	0.899
DarkNet53 + ECA	0.96	0.984	0.916
Ours (DarkNet53 + MSSA + ECA)	**0.98**	**0.995**	**0.937**

Note: Bold is the best result.

**Table 4:** Comparison experiment of recognition accuracy of different kinds of goods on various algorithms.

Algorithm Models	Bottle Shaped	Can Shaped	Bag Shaped
EfficientDet	84.74	88.23	76.94
CenterNet	81.31	62.39	61.87
SSD	82.14	84.23	74.18
Faster-RCNN	82.56	65.60	71.49
RetinaNet	88.47	81.04	74.17
YOLOv5	91.13	83.43	80.57
YOLOX	91.77	96.43	84.62
Ours	**94.64**	**96.80**	**91.42**

Note: Bold is the best result.

**Table 5:** Comparison experiment of recognition accuracy of occluded similar goods on various algorithms.

Classes of Similar Goods	Faster-RCNN	SSD	RetinaNet	YOLOv5	YOLOX	Ours
Large C’estbon	95.96	88.67	95.94	99.84	95.87	**99.91**
C’estbon	75.14	76.21	75.26	80.09	96.74	**97.62**
Sprite_Canned	59.76	83.54	78.91	81.48	96.44	**96.53**
Sprite_Bottled	90.92	93.36	90.37	**99.89**	96.87	97.46
Large Pepsi Cola	86.76	86.04	98.81	97.62	97.68	**98.94**
Pepsi Cola	83.11	77.64	88.60	97.24	82.79	**97.49**
Nongfu Spring	77.14	63.08	94.46	91.82	92.16	**95.27**
Little Nongfu Spring	62.74	84.09	74.18	**90.16**	89.62	87.46
**Average Accuracy**	78.94	81.58	87.07	92.27	93.52	**96.34**

Note: Bold is the best result.

## Data Availability

Data sharing not applicable.
